# Lipoxin A_4_ levels correlate with severity in a Spanish COVID-19 cohort: potential use of endogenous pro-resolving mediators as biomarkers

**DOI:** 10.3389/fimmu.2024.1509188

**Published:** 2025-01-23

**Authors:** Sergio Sánchez-García, Rafael I. Jaén, Roberto Lozano-Rodríguez, José Avendaño-Ortiz, Alejandro Pascual‐Iglesias, Laura Hurtado-Navarro, Eduardo López-Collazo, Lisardo Boscá, Patricia Prieto

**Affiliations:** ^1^ Departamento de enfermedades metabólicas e inmunitarias, Instituto de Investigaciones Biomédicas “Sols-Morreale”, Madrid, Spain; ^2^ Innate Immune Response Group, Instituto de Investigación del Hospital Universitario La Paz (IdiPaz), Madrid, Spain; ^3^ Microbiology Department, Hospital Universitario Ramón y Cajal and Instituto Ramón y Cajal de Investigación Sanitaria (IRYCIS), Madrid, Spain; ^4^ Centro de Investigación Biomédica en Red de Enfermedades Respiratorias (CIBERES), Instituto de Salud Carlos III, Madrid, Spain; ^5^ Centro de Investigación Biomédica en Red de Enfermedades Cardiovasculares (CIBERCV), Instituto de Salud Carlos III, Madrid, Spain; ^6^ Departamento de Farmacología, Farmacognosia y Botánica, Facultad de Farmacia, Universidad Complutense de Madrid, Madrid, Spain

**Keywords:** Covid, pulmonary disease, cytokine storm, lipoxin, biomarker

## Abstract

**Background:**

SARS-CoV-2, the causative virus of the COVID-19 global pandemic, leads to a wide variety of responses among patients. Some of them present a very severe phenotype, while others only experience mild symptoms or are even asymptomatic. This differential prognosis is tightly related to the inflammatory status of the patient. Although WHO declared the end of the emergency, the pandemic caused a great socio-sanitary impact in all countries. Thus, the possible outbreak of new biological diseases in the future makes it necessary to deepen the knowledge of this uncontrolled immune response and look for reliable biomarkers to help us predict its potential health impact. Specialized pro-resolving lipid mediators (SPMs) as lipoxins are endogenous mediators synthesized from arachidonic acid in the resolution stage of any inflammatory process. These lipids have pro-resolving actions in several pathological models, including reducing NF-κB-mediated inflammation, and inducing the antioxidant response through the Nrf-2 pathway. Thus, although a potential relationship has already been suggested between low levels of SPMs and COVID-19 severity, their true role as a predictive biomarker is still unknown.

**Methods and results:**

In this study, we have analyzed by ELISA the serum levels of lipoxin A_4_ (LXA_4_) in a representative Spanish cohort. We found reduced levels in deceased patients when compared to mild or severe patients, concomitant with a decrease in the LXA_4_ biosynthetic pathway and an increase in its degradation pathway. Furthermore, we have studied the correlation between the levels of this SPM and several pathology indicators, finding a significant correlation between increased LXA_4_ levels and a better prognosis of the patients.

**Conclusion:**

We propose to measure systemic LXA_4_ as a new promising biomarker to predict the survival in patients affected by SARS-CoV-2 and presumably to other viruses that can affect humanity in the future.

## Introduction

In December 2019, the expansion of a new viral pathology called “Severe Acute Respiratory Syndrome Coronavirus 2” (SARS-CoV-2) began, causing the Coronavirus Disease 2019 (COVID-19) (1, 2). This virus rapidly spread, becoming a pandemic disease affecting all countries around the world in 2020, causing millions of deaths. Even though the WHO has recently considered that the emergency is over, further research is needed to enable us to address other potential threats in the future.

SARS-CoV-2 does not affect everyone equally. The spectrum of symptoms ranges from mild to severe, while some patients are infected but remain asymptomatic (3). The most frequent symptoms are fever, cough, myalgia, fatigue or dyspnea. Moreover, this virus can also affect different organs, causing headache, diarrhea or even circulatory and cardiac complications, although the lungs are the main organ affected (4). As the disease progresses, some patients gradually develop an acute respiratory distress syndrome that, in the worst cases, can lead to multiple organ failure, compromising the survival of the patient (5).

At the cellular level, SARS-CoV-2 initially interacts with epithelial AT2 cells in the upper respiratory tract before reaching the lower lung epithelium, due to their high expression of the viral receptor ACE2 (6, 7). Upon infection, SARS-CoV-2 provokes an immune response that drives the production of pro-inflammatory cytokines (8). In fact, one of the most severe responses in patients corresponds to the uncontrolled release of pro-inflammatory cytokines, in a process known as “cytokine storm” or cytokine release syndrome (CRS), which has been related to the severity of the disease (9). This is mainly characterized by a high quantity of IL-6 and TNF-α but, depending on the cohort studied and the severity of the disease, patients can present higher levels of IL-1, IL-2, IL-4, IL-7, IL-10, CXCL10, CCL2, or IFN-γ, among others. Several meta-analyses have shown increases in pro-inflammatory cytokines and chemokines in patients, although their quantities vary among the different studies that have been published to date (10–12). Furthermore, other biomarkers have also been identified in the blood of COVID-19 patients, such as inflammatory (C-reactive protein; CRP), hematological (ferritin and D-Dimer) or tissue damage (LDH and lactate) markers, all of them related to an uncontrolled inflammatory response that leads to a worse prognosis (13–15).

Inflammation is a tightly regulated process in which several stages occur successively to eliminate the damage and allow for the recovery of tissue homeostasis. Thus, the inflammatory process is key in COVID-19 infection (16, 17). Once the damage agent has been blunted, the resolution stage initiates, inducing a switch between pro- to anti-inflammatory mediators (18). Lipoxins, as members of the specialized pro-resolving lipid mediators (SPMs), are generated in this stage to induce the end of the inflammatory response (19). Among lipoxins, lipoxin A_4_ (LXA_4_) was the first discovered and has been the most studied until now. These bioactive autacoids are endogenously produced by 5-, 12- and 15-lipoxygenases (ALOX) from arachidonic acid, requiring the interaction of different cell types (20, 21). Lipoxins exert their main actions through a specific receptor called FPR2 or ALXR (i.e. lipoxin receptor) (22), although they can also interact with others, such as AhR, CysLT1 or GPR32, driving less-known effects (23). From their discovery in the 80’s, many groups have described their pro-resolving actions, such as the inhibition of chemotaxis of neutrophils, induction of efferocytosis, modulation of macrophage survival and functioning, inhibition of the release of pro-inflammatory cytokines mediated by NF-κB and reduction of oxidative stress levels by Nrf-2 activation (24–26). Once these SPMs have fulfilled their function, they are rapidly removed by several enzymes, most notably 15-PGDH, which finalizes the resolution of the inflammatory process and the recovery of homeostasis (21, 27).

Thanks to their pro-resolving actions, the protective role of lipoxins has been demonstrated in pathological models *in vivo*, showing promising beneficial effects in different diseases such as periodontitis (28, 29) or arthritis (30, 31). Regarding the respiratory system, LXA_4_ reduces pulmonary inflammation and bronchial hyper-responsiveness inducing an improvement in asthma (32–34), acute lung injury (ALI) (35, 36), and ARDS (37). However, very little is known about the involvement of lipoxins in the pathology of COVID-19, although many studies have speculated about it (38, 39). Given their protective function in the lungs, their role in COVID-19 deserves further attention. Although it appears evident that lipoxin levels fluctuate in COVID-19 patients, it is still uncertain whether there is a correlation with the severity of the disease (40).

Due to the high variability of individual responses to this virus, the discovery of additional biomarkers is needed to help in the identification of patients with a bad prognosis. In this report, we have collected samples from 42 COVID-19 patients in the first 2 days since their admission to the emergency department (ED) of La Paz University Hospital in Madrid (Spain), as well as from 38 healthy volunteers. Patients were followed up until *exitus* or discharge and categorized according to their outcome. We have performed a comparative analysis incorporating clinical data and plasma levels of soluble molecules (cytokines, chemokines, and LXA_4_) in patients to determine if LXA_4_ levels can be considered as a reliable biomarker for COVID-19 severity. Thus, knowing in advance which patients can develop a severe symptomatology would allow us to offer treatments in the initial stages of the pathology, preventing the progression of the disease. Moreover, even though several therapeutic approaches have been used to treat COVID-19 in the last years, in some cases they remain ineffective in stopping the severe inflammatory phenotype and there is still a high incidence of patients that suffer long-term complications, known as “long COVID”, which has a strong inflammatory component (41, 42). Furthermore, despite the reduced mortality due to high vaccination rates, the emergence of new variants around the world has emphasized the need to deepen our understanding of the modulation of inflammatory pathways, demonstrating that a better understanding of this infection can help address others, which could affect us in the future.

## Materials and methods

### Patient cohort and sampling

In collaboration with López-Collazo´s lab, plasma samples were collected from 42 COVID-19 patients and 38 healthy volunteers upon admission (0-2 days) to the emergency department (ED) of La Paz University Hospital, before any treatment. Patients recruited were followed up until *exitus* or discharge and categorized according to their outcome: mild (outpatients + hospitalized with no O_2_ requirement, n = 14), severe (hospitalized with O_2_ requirement, n = 16) and *exitus* (deceased patients, n = 12; 28‐day mortality according to WHO U07.1 code). The cohorts were recruited from April 26 to November 20, 2020, during the first two waves of COVID-19 in Madrid, Spain.

Patients were included when they tested positive for SARS-CoV-2 by real-time quantitative polymerase chain reaction (RT-qPCR) from nasopharyngeal swabs before their hospitalization at La Paz University Hospital in Madrid (Spain). Patients with immunodeficiency (primary or acquired) were excluded from the study. Participants signed an informed consent and data were anonymized before study inclusion. Values of CRP, D-Dimer, LDH and lactate were obtained in the routine clinical blood analysis when patients were admitted to the emergency department.

### Plasma collection

Fresh blood from venipuncture was collected in lithium heparin and K2 ethylenediaminetetraacetic acid (EDTA) anticoagulant tubes (Vacuette^®^, Greiner Bio One, Kremsmünster, Austria). Blood was centrifuged and plasma isolated, aliquoted at 250 µl, and frozen at − 80°C until use.

### Lipoxin A_4_ levels determination

LXA_4_ levels were measured using a commercial ELISA kit (Neogen #407010) according to the manufacturer’s instructions. Firstly, an extraction step was carried out. Briefly, 50 μl plasma were mixed with methanol and sterile water, then acidified to pH 3.5 with 1M HCl. The extraction columns (Waters #WAT023501) were activated with methanol followed by washing with water. Then, samples were applied into the column, which was washed with water and hexane. After that, LXA_4_ was eluted using methyl formate. The solution was evaporated with a stream of N_2_ and then reconstituted with 250 μl ELISA extraction buffer.

For the ELISA assay, 50 μl of each extracted sample were analyzed in triplicates. Absorbance was measured at 650 nm in a Synergy HT microplate reader (Biotek) and sample values were interpolated with an LXA_4_ standard curve. An internal standard using commercial LXA_4_ (Cayman Chemical #90410) at 0.8 ng/ml was added to validate the ELISA kit.

### Cytokine levels determination

Measurements of cytokines were performed as described in (43). The bead-based multiplex assays were used (LEGENDplex Human Inflammation Panel 1 (12-plex: IL-1β, IL-2, IL-4, IFN-γ, TNF-α, MCP-1 (CCL2), CXCL10, IL-6, IL-8 (CXCL8), IL-10, IL-12p70 and IL-17A) (BioLegend) according to the manufacturer’s instructions.

### RNA isolation and qPCR

Human PBMCs were obtained from the patient’s whole blood after centrifugation at 250g for 5 min. RNA was isolated using QIAzol Lysis Reagent (Qiagen), followed by reverse transcription of 250 ng RNA into cDNA using the High-Capacity cDNA Reverse Transcription kit (Applied Biosystems #4368813). The PCR reaction was carried out at 2.5 ng/µl using *Power* SYBR Green PCR Master Mix (Applied Biosystems #4367659) and the pertinent primers (see **Table 1**) at 250 nM in a 7900HT Fast real-time PCR system (Applied Biosystems). Fold Induction was determined by ΔΔCt method using the ribosomal *RPLP0* gene as endogenous reference.

**Table 1:** List of primer forward and reverse sequences for each gene used in RT-qPCR assays.

Gene	Forward sequence	Reverse sequence
*ALOX5*	TACTTGTCCCCAGACCGGAT	AGATGTGACTGCCAAGAGGC
*ALOX12*	AAGCCCAGCTGCATAGAGAA	TGGGGGAGGAAATAGAGCCTT
*ALOX15*	AGTGTGGCCATCTAAGCGTC	GGCAGGGCTATAACCACGAA
*FPR2*	AGTCTGCTGGCTACACTGTTC	TGGTAATGTGGCCGTGAAAGA
*HPGD*	ATGCACGTGAACGGCAAAG	ATCCAGGGCAGCTTTACACT
*RPLP0*	CAGGCGTCCTCGTGGAAGTGAC	CCAGGTCGCCCTGTCTTCCCT

### Statistical analysis

All values shown on graphs represent mean ± standard deviation (SD). Statistical difference of means between data groups was determined using one-way ANOVA followed by Tukey’s range test. Correlations were evaluated by two-tailed Pearson’s analysis. Receiver operating characteristic (ROC) curve analysis was used to define whether LXA_4_ could be used as a predictor of mortality. The optimal cut-off value was estimated using the Youden index. A *p-value* ≤ 0.05 was considered statistically significant. All analyses were realized using GraphPad Prism Software.

## Results

To ameliorate the therapeutic approaches of COVID-19 patients, it is essential to establish new parameters that allow us to improve their prognosis. Since it has been demonstrated that the development of an exacerbated inflammatory response is one of the main factors contributing to the worsening of the disease, it is important to discover inflammatory biomarkers that allow us to assess the evolution of patients, avoiding the development of the most severe phenotypes. For this purpose, we classified our cohort depending on the outcome in 3 experimental groups: 14 patients as mild (outpatients + hospitalized with no O_2_ requirement), 16 patients as severe (hospitalized with O_2_ requirement) and 12 *exitus* (deceased). All the patients’ samples were from days 0-2 from admission to the ED, which allowed us to do a prospective study from an early inflammatory status. We also recruited 38 healthy volunteers (HV). The demographic data for the diseased patients can be found in **Supplementary Table S1**. Firstly, we measured the plasmatic levels of some damage markers that can be clinically considered as biomarkers for a worse progression of COVID-19 (**Table 2**). Thus, elevated serum levels of CRP, a widely used clinical biomarker of systemic inflammation, have also been associated with severe viral infection. In our cohort, severe patients already had elevated CRP values, above the established threshold of 50 mg/l, while those who died showed higher values (Mean ± SD = 114.7 ± 116.6 mg/l). Another clinical parameter that has been useful as a biomarker of the severity of COVID-19 is D-dimer, a fibrin degradation product that is associated with thrombosis in patients. We detected also significant higher levels in patients with severe disease (Mean ± SD = 1.14 ± 0.91 µg/ml) and deceased patients (Mean ± SD = 5.94 ± 9.3 µg/ml). Furthermore, LDH is a metabolic enzyme found in almost every cell in the body. The increase of LDH in the blood reflects tissue destruction and, in the COVID-19 context, is a sign of lung damage caused by viral pneumonia. Levels of this marker above 245 U/l are considered a bad indicator of the progression of the disease. All the COVID-19 patients analyzed exceeded those levels, but they were especially increased in the severe and *exitus* groups. Concordantly, lactate, which is the product of the LDH-mediated reaction, showed a significant increase in the serum levels of *exitus* group, although they did not reach the conventional hyperlactatemia values observed for other diseases (>2 mmol/l). Ferritin was the last biomarker analyzed in this study, whose elevated levels in COVID-19 patients (≥287.4 ng/ml) are commonly associated with cytokine storm (44). Interestingly, in our cohort, the ferritin levels were higher than this value in severe and *exitus* patients, according to those obtained in the other damage markers. All these data indicate a strong inflammatory background, which increases as the severity escalates, thus supporting our patient stratification based on disease severity.

**Table 2:** Quantification of damage biomarker levels in serum of COVID-19 patients, classified based on disease severity.

BIOMARKER	MILD (14)	SEVERE (16)	*EXITUS* (12)
**CRP (mg/l)**	48.0 ± 59.5	95.6 ± 93.8	114.7 ± 116.6
**D-dimer (μg/ml)**	0.66 ± 0.41	1.14 ± 0.91*	5.94 ± 9.3*
**LDH (U/l)**	266.9 ± 95.1	370.6 ± 104.9 **	433.3 ± 208.8***
**Lactate (mmol/l)**	1.04 ± 0.26	1.11 ± 0.5	1.86 ± 0.95**
**Ferritin (ng/ml)**	176.2 ± 129.4	548.0 ± 615.4	859.2 ± 815.9*

Values represent mean levels ± SD of serum CRP (mg/l), D-dimer (mg/ml), LDH (U/l), lactate (mmol/l) and ferritin (ng/ml), measured in our COVID cohort, and classified depending on the severity. *p ≤ 0.05, **p ≤ 0.01, ***p ≤ 0.001 versus mild group.

Furthermore, to deepen in the inflammatory profile of the cohort, we also analyzed if they had developed a “cytokine storm”-like phenotype. For this purpose, we quantified the levels of 10 cytokines that have been described to be increased in this pathology (**Table 3**). Our results showed that most of the pro-inflammatory cytokines analyzed (IL-1β, IL-2, IL-6, IL-8, IFN-γ, TNF-α, CCL2, CXCL10) had increased levels in severe or *exitus* patients compared to those with mild symptoms. Due to the high variability of data, this increase was only significant in the *exitus* group for IL-6, IL-8, CCL2 and CXCL10. Moreover, we determined the levels of IL-4 and IL-10, which are classically considered as anti-inflammatory mediators. Although these cytokines are also increased in some cohorts, both cytokines showed similar levels in all patients, independently of their severity.

**Table 3:** Quantification of several cytokine levels in serum from COVID-19 patients, classified based on disease severity.

CYTOKINES (pg/ml)	MILD (14)	SEVERE (16)	*EXITUS* (12)
**IL-1β**	0.5 ± 1	1.3 ± 2.7	0.7 ± 2.3
**IL-2**	0.6 ± 1.4	1.3 ± 2.2	3.3 ± 6
**IL-4**	4.3 ± 8.5	6.6 ± 10	1.9 ± 4.8
**IL-6**	14.3 ± 23.8	68.4 ± 103.9	231.8 ± 319*
**IL-8 (CXCL8)**	3.3 ± 7.5	8.5 ± 11.8	50.7 ± 82.1*
**IL-10**	2.1 ± 3.9	7.8 ± 10.3	6.8 ± 9.2
**IFN-γ**	9.3 ± 17.2	14.5 ± 17.4	575.7 ± 1790.1
**TNF-α**	0.8 ± 2	4 ± 9.1	1.4 ± 4
**MCP-1 (CCL2)**	96.3 ± 66.1	170.4 ± 142.5	406.5 ± 460.1*
**IP-10 (CXCL10)**	176.2 ± 145.7	709.1 ± 765.6	1289.6 ± 1763*

Values represent mean levels ± SD of IL-1β, IL-2, IL-4, IL-6, IL-8, IL-10, IFN-g, TNF-a, CCL2 and CXCL10, measured in our COVID-19 cohort, and classified depending on the severity. *p ≤ 0.05 versus mild group.

**Figure 1:**
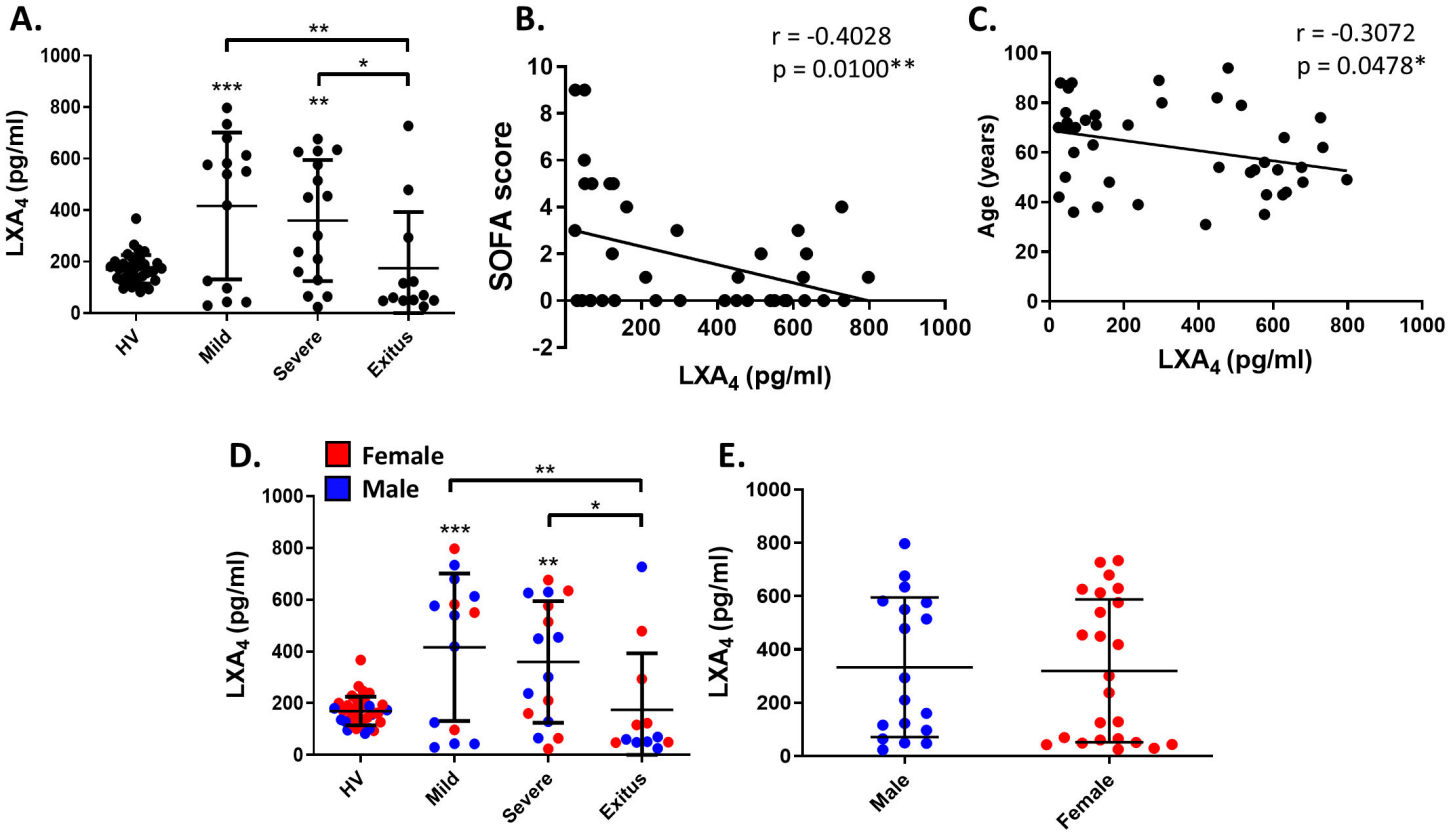
Analysis of lipoxin A_4_ serum levels in our cohort of COVID-19 and its correlation with SOFA score, gender and age. **(A)** Distribution of LXA_4_ levels in the serum of patients classified according to the degree of COVID-19 severity (mild, severe or *exitus*), compared with healthy volunteers (HV). Correlation analysis between LXA_4_ levels and SOFA score **(B)**, gender **(C, D)** or age **(E)**. Each symbol corresponds to a different individual. In **(D, E)**, red dots correspond to females and the blue ones to males. In **(B, C, E)**, healthy volunteers are not included. *p ≤ 0.05, **p ≤ 0.01, ***p ≤ 0.001 versus the indicated group. The values in panels **(B, C)** correspond to Pearson’s *r* and *p* value, respectively.

Once the pro-inflammatory profile of our patients was completed, we were interested in analyzing whether the resolution phase had occurred properly. Low levels of LXA_4_ in human serum have been associated with higher disease severity in many different pathologies (45–47), mainly due to a defective resolution of inflammation. Therefore, we sought to elucidate its relevance in COVID-19 patients. To do this, we analyzed the levels of LXA_4_ in serum samples from our cohort (**Figure 1A**). Compared to HV, whose mean ± SD levels were 169.8 ± 55.2 pg/ml, both mild and severe groups of patients showed significantly higher levels of this pro-resolving mediator (416.4 ± 284.8 pg/ml and 359.8 ± 234.9 pg/ml, respectively). Interestingly, those patients who died (*exitus* group) presented significantly lower levels of LXA_4_ (174.3 ± 219 pg/ml) than mild and severe ones, indicating that failure in this pro-resolving response could be behind the worsening of these patients. To further explore this hypothesis, we compared levels of LXA_4_ with the SOFA score of the patients, which is used to assess the severity of patients based on the function of various organs. Accordingly, LXA_4_ levels were negatively correlated to the SOFA score (**Figure 1B**). Moreover, to study the potential influence of age or sex in LXA_4_ levels, we determined the correlation between these factors. Thus, we observed that lipoxin levels significantly decrease with age (**Figure 1C**), which can be related to a less effective resolution in older people. However, levels of this SPM are independent of gender, showing similar quantities in both males and females (**Figures 1D, E**). In addition, we also determined if there was any correlation with other demographic characteristics like smoking, hypertension or obesity, but we did not observe any significant differences (**Supplementary Figure S1**). Moreover, when we compared lipoxin levels in our cohort with the molecular biomarkers shown in **Table 2**, we observed a clear negative correlation with all of them (**Supplementary Figure S2**).

**Figure 2:**
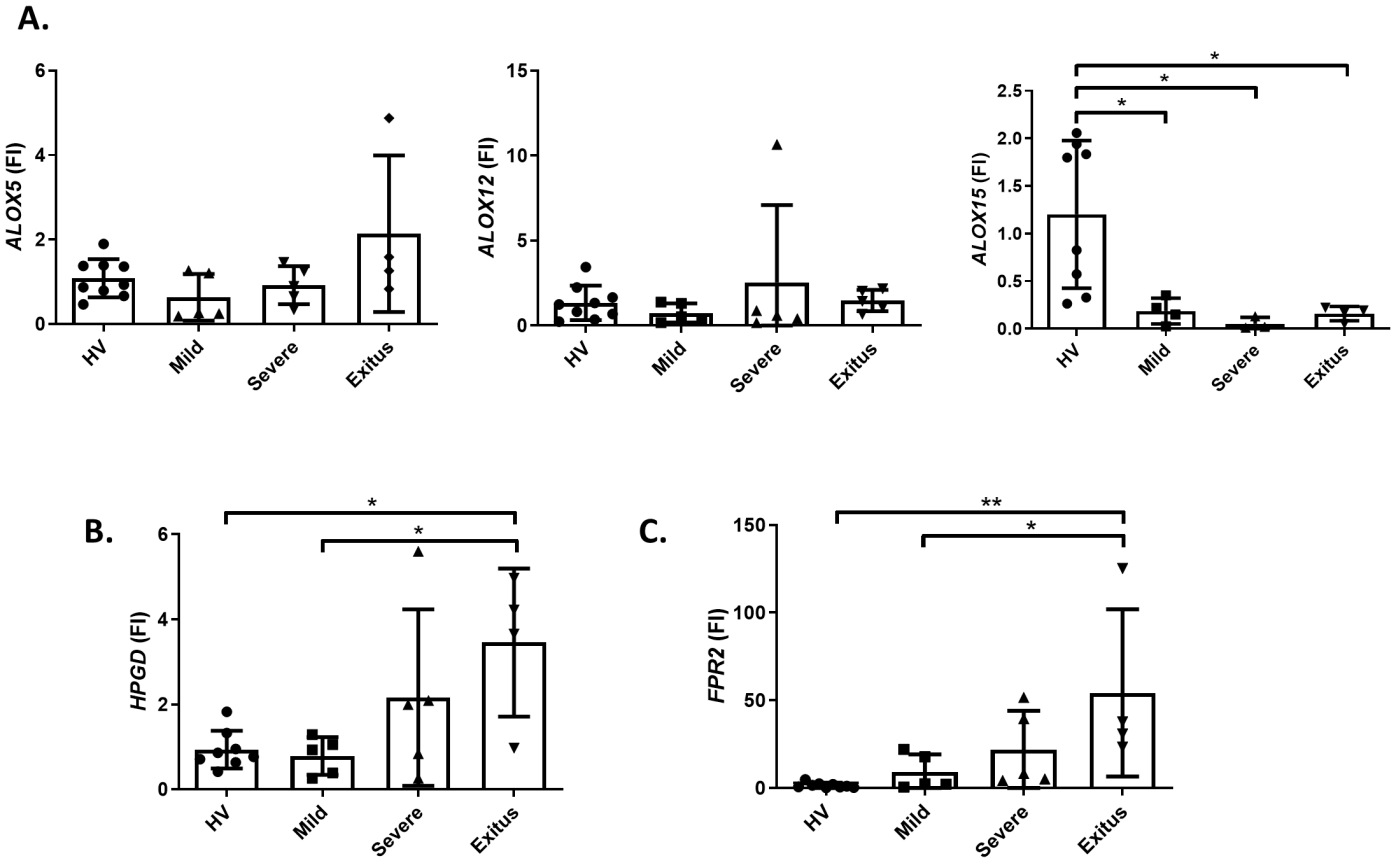
RNA expression of genes implicated in the synthesis or degradation of lipoxin A_4_ in isolated PBMCs from patients and healthy donors. mRNA levels of *ALOX5, 12* and *15* genes corresponding to the 5-, 12- and 15-lipoxygenases **(A)**, *HPGD* (15-PGDH) **(B)** and *FPR2* (ALXR) **(C)**, were quantified in PBMCs from healthy volunteers and COVID-19 patients classified depending on the disease severity. *RPLP0* was used as endogenous reference. *p ≤ 0.05, **p ≤ 0.01 versus the indicated group.

Since our data suggest that LXA_4_ levels can be modulated depending on the COVID-19 severity, we next determined the levels of the main enzymes involved in LXA_4_ synthesis and degradation pathways. For that, we analyzed the mRNA levels of *ALOX5, 12 *and *15* (implied in biosynthesis) and *HPGD* (which encodes for the main enzyme responsible for its degradation) in blood-derived PBMCs obtained from patients (**Figure 2**). Regarding the lipoxygenase biosynthetic pathway, we did not detect changes in the levels of *ALOX5* or *12* between the different severity groups, unlike *ALOX15*, which was repressed in all COVID-19 patients, regardless of their severity (**Figure 2A**). Furthermore, the levels of *HPGD* were upregulated in the deceased compared to the mild group and healthy volunteers (**Figure 2B**), indicating that the lower levels of LXA_4_ detected in this group can be due to higher levels of degradation. Interestingly, when we quantified the levels of the main lipoxin receptor, *FPR2* (which encodes for ALXR), we observed that it was positively correlated with disease severity and only became significant in deceased patients (**Figure 2C**).

**Figure 3:**
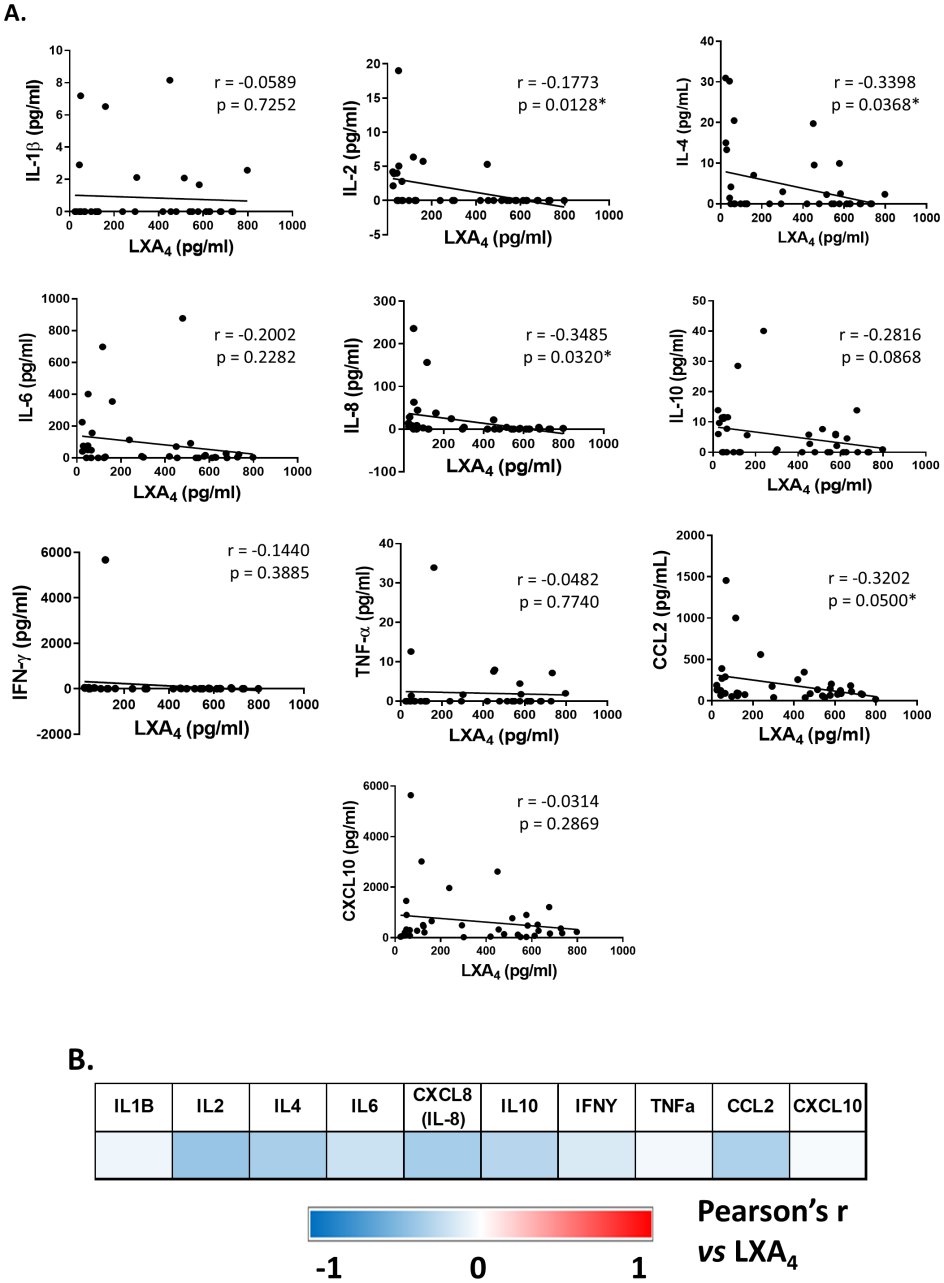
Correlation analysis between lipoxin A_4_ and inflammatory-related cytokines serum levels. The shown values correspond to *r* and *p values* of Pearson’s *r* coefficient for each cytokine *versus* LXA_4_ levels **(A)**. A stronger color intensity corresponds with a higher correlation index **(B)**. *p ≤ 0.05 versus the indicated group.

Subsequently, we analyzed if LXA_4_ levels were related to the inflammatory profile in all patients of our cohort, performing a correlation analysis between LXA_4_ levels and the main cytokines implied in the inflammatory response. Although some variability was detected between patients, our results showed that lipoxin levels negatively correlated with IL-2, IL-4, IL-8, and CCL2 (**Figure 3A**). This relation can be seen in **Figure 3B**, where a higher intensity of blue color corresponds with a stronger correlation.

**Figure 4:**
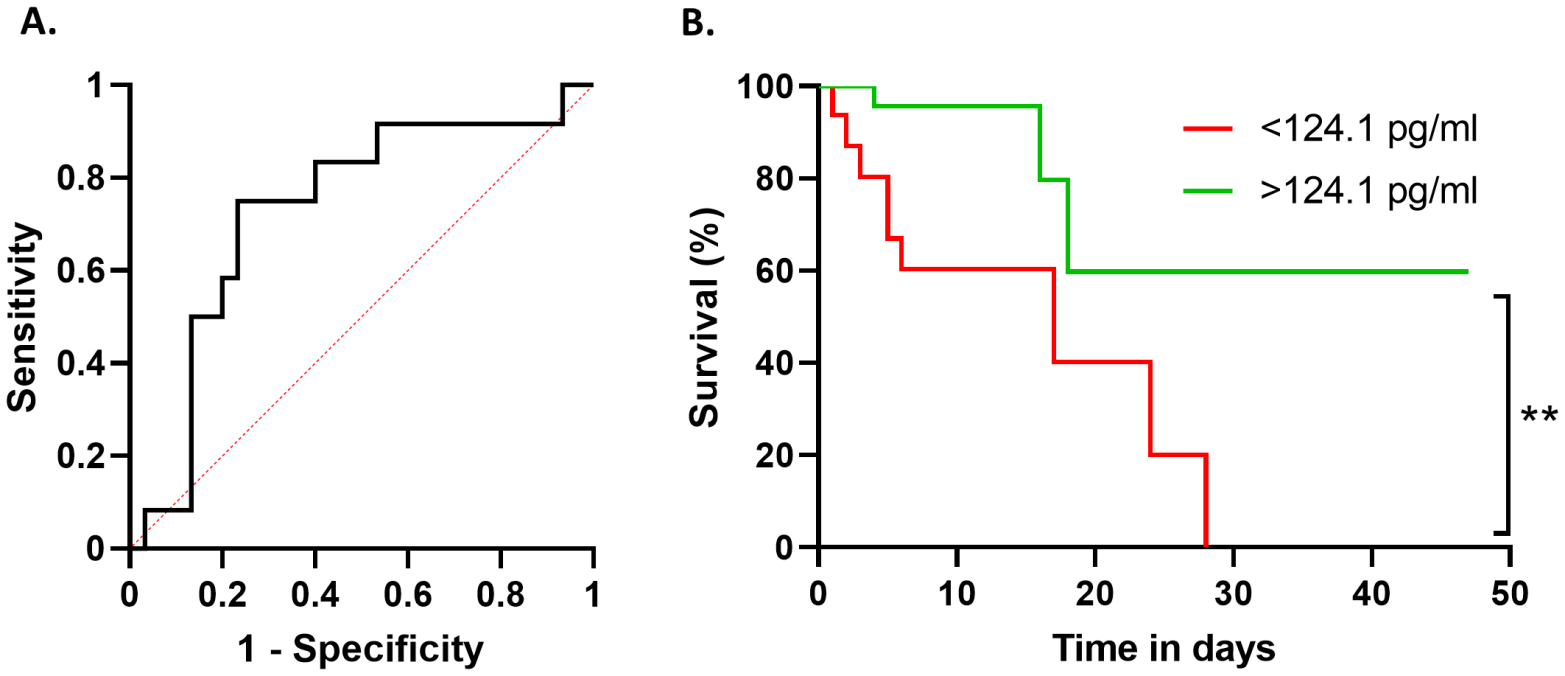
Prognostic ROC curve to assess if serum levels of LXA_4_ can be considered as a biomarker of survival probability. ROC curve showing the sensitivity and 1-specificity values for each cut-off point given by the Youden analysis **(A)**. Comparison between the probability of survival of patients above (green line) or below (red line) the selected cut-point of 124.1 pg/ml of LXA_4_ **(B)**. **p ≤ 0.01 versus the indicated group.

Finally, we evaluated whether LXA_4_ could be a potential biomarker for COVID-19. For this purpose, we used an Area Under the Curve/Receiver Operating Characteristic (AUC/ROC) analysis (**Figure 4**), which showed an AUC/ROC of 0.7306. Using the Youden index, we selected an LXA_4_ serum level of 124.1 pg/ml, which yielded a sensitivity of 0.75 and a specificity of 0.77. The comparison between both survival curves was performed using a Log-rank (Mantel-Cox) test, which showed a statistically significant difference (*p* = 0.0046). These data provide insightful results regarding LXA_4_ diagnostic value: the finding that patients with serum LXA_4_ levels above 124.1 pg/ml have a higher and statistically significant probability of survival suggests that LXA_4_ could serve as a meaningful indicator for patient prognosis. In this case, it has been shown that LXA_4_ levels can effectively discriminate between patients with different survival outcomes, indicating high diagnostic accuracy.

## Discussion

SARS-CoV-2 is a virus that caused a global pandemic in 2020, resulting in millions of deaths worldwide due to COVID-19. Despite the current lower severity of the pathology, there still exists a subset of individuals who experience severe manifestations, as well as those who have developed long-term symptoms, known as ‘long COVID’ (41). Furthermore, the unprecedented crisis we faced underscored the importance of developing biomarkers of injury, which could serve as potential diagnostic or prognostic tools against future potential threats.

Several serum biomarkers have been already proposed to stratify patients. Among them, there are biochemical markers such as LDH and its product lactate, coagulation markers like D-dimer, or inflammatory markers like CRP and ferritin (48, 49). Data obtained from recent metanalysis have established threshold values for all of them, above which, these parameters positively correlate with increased disease severity. In our cohort, both LDH and D-dimer levels were significantly augmented in severe and *exitus* patients, which could be related to thrombosis and lung damage in those patients (50–52). Moreover, deceased patients showed higher levels of ferritin and lactate, according to a higher inflammatory response in the severe patients. Interestingly, although CRP levels also increased with severity, they did not reach statistical significance. This may be because, as a marker of systemic inflammation, it is much more variable among the individuals included in the study. Beyond molecular damage mediators, it has also been described that in some patients a “cytokine storm” can be developed, inducing the systemic release of a high number of pro-inflammatory cytokines and chemokines into the bloodstream, worsening the prognosis of those affected (9). Concordantly, in our cohort, more critical patients presented higher levels of most of the pro-inflammatory cytokines, although it was only significant for IL-6, IL-8, CCL2 and CXCL10 due to the high variability between patients. Both IL-6 and IL-8 have been broadly considered as prognostic biomarkers in COVID-19. Increased levels of these cytokines are related to an exacerbated inflammatory response and, therefore, to a worse clinical outcome (53). Moreover, it has been described that an increase in IL-6 is related to thrombosis induction and CRP release (54). Beyond cytokines, some chemokines are also implicated in the severity of COVID-19, acting as chemoattractants for immune cells, such as CCL2 or CXCL10, which contribute to the amplification of the inflammatory response (55). The negative correlation between LXA_4_ and all these pro-inflammatory biomarkers supports the suitability of LXA_4_ as a prognostic biomarker. According to previous data (43), there is high variability between patients in systemic cytokine levels of our cohort. This can be associated with several factors, such as genetic polymorphisms, comorbidities, viral load or health personal status. It can also vary depending on the different treatments received by the patients. Thus, the variability in inflammatory cytokine levels in serum among COVID-19 patients is influenced by a complex interplay of factors.

In contrast, emerging research has also highlighted the potential role of SPMs as potential biomarkers in COVID-19. In fact, lipidomic studies of BALs of severe COVID-19 patients versus healthy donors found increased levels of pro-inflammatory COX metabolites and leukotrienes, and also extensive levels of some SPMs such as D-series resolvins and protectin D1 (56). Additionally, lipoxins, which play a fundamental role in the resolution of inflammation, not only appear to be good indicators of the resolution phase of inflammation, but also offer insights into the dynamics of the immune response and the severity of the disease (57). Thus, it has been suggested that SPMs could directly inactivate enveloped viruses, including SARS-CoV-2 (58). In our cohort, we observed that, while mild or severe patients had higher levels of LXA_4_ than healthy volunteers, this mediator was reduced in those who died. Elevated levels of this SPM detected in patients with an intermediate degree of severity of the pathology may be related to the body’s attempt to stop the inflammation occurring after viral infection. However, this mechanism appears to fail in those more severe cases, hence the levels are lower in those who ultimately died from COVID-19 indicating a failing resolution capacity. Indeed, most of the deceased patients had even lower lipoxin levels than the HV group, suggesting that not only is lipoxin biosynthesis impaired, but its degradation could also be enhanced. This correlation of lipoxin levels with disease severity agrees with previous studies in other pathologies, such as stroke (59, 60), heart failure (61) and asthma (34). Consistent with previous reports (62), these results do not depend on sex, being equivalent in both genders. However, LXA_4_ serum levels decline with age, showing lower resolutive capacity in older people, which has been related to cognitive impairment (63). These results agree with an observational pilot study that was performed on adult patients hospitalized due to non-severe COVID-19, which showed that serum lipoxin levels increase 24-48h after hospitalization, being this increase lower in older patients (64). To explain these changes in LXA_4_ levels, we analyzed molecular pathways involved in both the production and clearance of SPM in PBMCs isolated from patients, detecting significantly lower levels of 15-lipoxygenase in all patients compared to healthy donors, independently of the severity. Since this enzyme catalyzes not only the oxygenation of arachidonic acid mainly to 15-HETE to produce different SPMs, but also metabolizes EPA and DHA to induce the formation of ω3-derived SPMs (65), its inhibition can directly explain a failure in the resolutive response. This is also supported by higher levels of 15-PGDH in the most severe patients, indicating higher rates of degradation of LXA_4_. Interestingly, we also detected higher levels of ALXR expression in those patients with more critical disease in our study population, which may be related to the body’s attempt to improve the signaling in response to low systemic levels of SPMs, enhancing the main signaling pathway driven by LXA_4_.

Furthermore, all these cytokines and damage biomarkers are inversely correlated to LXA_4_ levels. This may indicate that patients with higher severity levels not only have an excessive release of pro-inflammatory cytokines, which accentuate the immune response but also possess low levels of pro-resolving mediators such as lipoxins. This contributes to the inability to adequately resolve inflammation, causing it to become chronic and promoting greater tissue damage. If this affects the lungs or other vital organs, it can end up endangering the patient’s life. In this sense, the AUC/ROC curve analysis supports the potential of LXA_4_ as a prognostic biomarker for COVID-19, as does the Youden index that establishes the specific threshold of 124.1 pg/ml for serum levels. Patients above this threshold are more likely to survive, which could help clinicians make informed decisions about patient management and treatment strategies. Identifying a reliable biomarker for COVID-19 severity is essential for improving patient outcomes. Although ELISA is a widely used technique, there are valid concerns regarding its ability to accurately measure SPMs in plasma, which may be considered as a limitation of this study. A recent report indicated that ELISA lacks the specificity required to quantify SPM in biological samples, suggesting that only “high-performance” liquid chromatography coupled with mass spectrometry (HPLC-MS) can reliably achieve this (66). Nevertheless, we believe that ELISA remains a reliable and valuable technique for detecting LXA_4_, as confirmed by adding commercially available internal LXA_4_ standards. In fact, many recent publications continue to employ ELISA for SPM quantification (67–69) mainly because it offers a reproducible and accessible method, while HPLC-MS is less readily available. Thus, if LXA_4_ levels can be consistently linked to survival rates, they could be incorporated into routine clinical assessments to stratify patients based on their risk and tailor interventions accordingly. Furthermore, this finding opens the door to performing the same analysis in other pathologies in which lipoxins have been shown to play a key role, such as COVID-related myocarditis, asthma or periodontitis. As LXA_4_ is known to have anti-inflammatory properties, its elevated levels in survivors might indicate a more effective resolution of inflammation, which is a critical aspect of recovering from severe COVID-19. In conclusion, measurement of LXA_4_ could help in assessing patient prognosis and guide therapeutic interventions aimed at promoting the resolution of inflammation and improving outcomes in patients with COVID-19.

## Data Availability

The raw data supporting the conclusions of this article will be made available by the authors, without undue reservation.

## Glossary

15-PGDH: 15-Hydroxyprostaglandin Dehydrogenase
ACE2: Angiotensin-Converting Enzyme 2
AhR: Aryl Hydrocarbon Receptor
ALI: Acute Lung Injury
ARDS: Acute Respiratory Distress Syndrome
AT2: Alveolar Type 2
AUC/ROC: Area Under the Curve/Receiver Operating Characteristic
CCL2: C-C Motif Chemokine Ligand 2
COVID-19: Coronavirus Disease 2019
CRP: C-Reactive Protein
CRS: Cytokine Release Syndrome
CXCL10: C-X-C Motif Chemokine Ligand 10
CysLT1: Cysteinyl Leukotriene Receptor 1
ED: Emergency Room
FPR2/ALXR: N-Formyl Peptide Receptor 2/Lipoxin Receptor
GPR32: G Protein-Coupled Receptor 32
HV: Healthy Volunteers
IL: Interleukin
IFN-γ: Interferon Gamma
LDH: Lactate Dehydrogenase
LXA_4_: Lipoxin A_4_
NF-κB: Nuclear Factor Kappa B
Nrf-2: Nuclear Factor Erythroid-Derived 2-like 2
SARS-CoV-2: Severe Acute Respiratory Syndrome Coronavirus 2
SPMs: Specialized Pro-Resolving Lipid Mediators
TNF- α: Tumor Necrosis Factor Alpha
WHO: World Health Organization.
