# Supplementary material for: Lipoxin A4 levels correlate with severity in a Spanish COVID-19 cohort: potential use of endogenous pro-resolving mediators as biomarkers
Source: Front Immunol. 2025 Jan 23;15:1509188. doi: 10.3389/fimmu.2024.1509188 (PMC11798798; doi:10.3389/fimmu.2024.1509188)
Supplement: Supplementary file 1 [file DataSheet1.docx]

**SUPPLEMENTARY LEGENDS**

| **DEMOGRAPHIC DATA** | **GROUPS** | **HEALTHY (38)** | **MILD (14)** | **SEVERE (16)** | ***EXITUS* (12)** |
| --- | --- | --- | --- | --- | --- |
| **GENDER** | MALE/FEMALE | 29/9 | 4/10 | 8/8 | 6/6 |
| **AGE** | CONTINUOUS | 51.29 ± 11.31 | 56 ± 16.15 | 57.5 ± 15.66 | 75.83 ± 14.38** |
| **SMOKER** | YES/NO | - | 1/13 | 3/13 | 5/7 |
| **OBESITY** | YES/NO | - | 2/12 | 5/11 | 0/12 |
| **HYPERTENSION** | YES/NO | - | 4/10 | 3/13 | 7/5 |
| **CORTICOIDS** | YES/NO | - | 2/12 | 12/4 | 8/2 (2) |
| **TOCILIZUMAB** | YES/NO | - | 0/14 | 1/15 | 1/9 (2) |
| **REMDESIVIR** | YES/NO | - | 3/11 | 8/8 | 2/8 (2) |
| **COVALESCENT PLASMA** | YES/NO | - | 0/14 | 2/14 | 0/10 (2) |
| **SOFA SCORE** | CONTINUOUS | - | 0.64 ± 1.5 | 0.88 ± 1.26 | 4.8 ± 2.82**** |

***Supplementary table 1****.* ***Demographic data of both healthy donors and COVID-19 patients***. For healthy donors, only age and gender data were disposable due to protective laws, and for COVID-19 patients, data from their gender, age, some health conditions as if they were smokers, hypertensive or obese, treatments received, and SOFA score were recorded in La Paz Hospital after admission. In the exitus group, the number within parenthesis indicates the number of missing data. **p ≤ 0.01, ****p ≤ 0.0001 *versus* mild group.

***Supplementary figure 1. Lipoxin A_4_ serum levels in patients according to inflammation and damage biomarkers***: smoker, hypertensive or obese. In each condition, the square represents negative and the circle positive.

***Supplementary figure 2. Correlation analysis between lipoxin A_4_ (pg/ml) and damage biomarkers serum levels***. **A**. Each dot represents a patient. Shown values correspond to Pearson’s correlation coefficient (*r*) and its p-value. **B**. The colors represent the corresponding Pearson’s *r* coefficient in patients versus lipoxin A_4_ levels. **p* ≤ 0.05 *versus* the indicated group.
